# The global, regional, and national burden of kidney cancer and attributable risk factor analysis from 1990 to 2017

**DOI:** 10.1186/s40164-020-00181-3

**Published:** 2020-09-29

**Authors:** Xianguang Bai, Ming Yi, Bing Dong, Xinhua Zheng, Kongming Wu

**Affiliations:** 1 Medical School of Pingdingshan University, Pingdingshan, Henan China; 2 Department of Oncology, Tongji Hospital of Tongji Medical College, Huazhong University of Science and Technology, Wuhan, Hubei China; 3 Department of Molecular Pathology, The Affiliated Cancer Hospital of Zhengzhou University and Henan Cancer Hospital, Zhengzhou, Henan China

**Keywords:** Kidney cancer, Social-demographic index, Global burden of diseases, Cancer epidemiology, Attributable risk factor

## Abstract

**Background:**

Kidney cancer’s incidence and mortality vary in different regions and countries. To compare and interpret kidney cancer’s burden and change trends in the globe and in different countries, we conducted this study to report the global kidney cancer burden and attributable risk factors.

**Methods:**

Data about kidney cancer’s incidence, death, disability-adjusted life-year (DALY) were extracted from the Global Burden of Diseases 2017. Besides, social-demographic index (SDI) values were adopted to investigate the correlation between kidney cancer’s burden and social development degrees.

**Results:**

In the globe, the incidence case of kidney cancer increased sharply from 207.31*10^3^ in 1990 to 393.04*10^3^ in 2017. High SDI countries had the highest kidney cancer’s burden with a decreased trend in incidence rate. On the contrary, the incidence rate was rapidly increased in low-middle SDI countries, although their burden of kidney cancer kept relatively low. At the same time, the deaths of kidney cancer increased from 68.14*10^3^ to 138.53*10^3^, and the kidney cancer-related DALYs increased from 1915.49*10^3^ in 1990 to 3284.32*10^3^ in 2017. Then, we searched the GBD database for kidney cancer-related risk factor. The high body-mass index and smoking were the main factors contributing to kidney cancer-related mortality.

**Conclusions:**

Generally, from 1990 to 2017, the incidence rate in developed countries had gone down from the historic peak values while the incidence rate was still on the rise in developing counties. Given the aging trend in the globe, it is necessary to appeal to the public to decrease the exposure of kidney cancer-associated risk factors.

## Background

Kidney cancer is a common genitourinary malignancy with poor prognosis. In 2019, kidney cancer was the sixth most commonly diagnosed cancer in males and the eighth in females in the United States [1]. In general, the incidence of kidney cancer predominates in males, and the ratio of male to female is approximately 1.5:1 [2]. Besides, the incidence rate of kidney cancer varies in different countries, which is commonly higher in developed countries than in developing countries [3]. Although the incidence keeps increasing, the relative survival rate of kidney cancer has been dramatically improved during the past three decades [4]. In the United States, the 5-year survival rate of kidney cancer increased from nearly 50% in the 1970s to 57% in the 1980s, then reached 73% in the 2000s [5]. The increasing understanding of kidney cancer’s molecular biology and cytogenetic propels the development of systemic treatment strategy. In the past decade, several targeted agents were developed and entered into clinical studies. Antiangiogenic agents such as Bevacizumab, Sorafenib, and Sunitinib have been approved for kidney cancer treatment [6, 7]. Besides, agents targeting the mTOR pathway, such as Temsirolimus and Everolimus, have also been approved for metastatic kidney cancer treatment [8].

Moreover, because kidney cancer cells tend to harbor frequent frameshift mutations, kidney cancer always has strong immunogenicity and is suitable for immunotherapy [9, 10]. Antibodies targeting programmed cell death 1 (PD-1) or PD-L1 such as Nivolumab, showed a potent and durable anti-cancer effect in kidney cancer patients [11]. Apart from a single agent, combination therapy is another promising treatment strategy. Immune checkpoint inhibitor combining antiangiogenic agents exhibited a synergistic effect for kidney cancer [12, 13].

Kidney cancer is a heterogeneous panel of tumors with various genetic alterations and molecular changes [14, 15]. Renal clear cell, chromophobe, and papillary carcinomas are the most commonly diagnosed solid renal cancers, which account for over 85% of all kidney malignancies [14]. Up to now, the mechanisms by which kidney cancer initiates and develops are still unclear. In most kidney cancer tissues, the structural changes in the short arm of chromosome 3 could be detected [16]. Over 80% of renal clear cell cancer patients harbor the genetic or epigenetic alteration of *VHL* (a cancer suppressor gene), which participates in oxygen sensing and proteasome degradation [17]. Besides, a group of other gene mutations, including *PBRM1*, *BAP1*, *SETD2*, and *TCEB1,* also play an essential role in kidney cancer initiation and development [18–21].

Moreover, some lifestyle risk factors, such as obesity and tobacco exposure, have been documented to increase the risk of kidney cancer [22, 23]. Smoking increased the risk of renal cell carcinoma by 50% in males and by 20% in females [24]. A rise of 5 kg/m^2^ in body mass index elevated the risk of renal cell carcinoma by 24% in males and 34% in females [25]. Besides, hypertension and low consumption of fruit or vegetable were related to the increased risk of kidney cancer [26, 27].

Although kidney cancer is a severe health threat in the globe, a comprehensive assessment of incidence, death, and disability-adjusted life-year (DALY) is not available until now. Based on the data from the GBD 2017 database, we estimated the global, regional, and national burden of kidney cancer from 1990 to 2017, which might be meaningful for policy-makers to allocate healthy resources rationally.

## Methods

### Data acquisition

Global Burden of Diseases 2017 (GBD 2017) database contains the burdens of 354 diseases in the globe, different geographic areas, and 195 countries and territories [28–32]. Data about kidney cancer’s incidence, death, DALY, as well as their corresponding age-standardized rates (ASRs), were downloaded by the Global Health Data Exchange (GHDx) (website: http://ghdx.healthdata.org/gbd-results-tool). In the meanwhile, the information about the distributions of sex and age was also acquired. SDI value is the average of total fertility, per capita income, and the years of education, which is developed to reflect social development degree. As previous studies documented, social development degree might affect the incidence and mortality of kidney cancer. Thus we obtained the social-demographic index (SDI) values to investigate the correlation between kidney cancer’s burden and social development degrees in different countries.

### Statistical analysis

The numbers of incidence, death, and DALY were the main parameters evaluating the burden of kidney cancer. To avoid the interferences of population change and age distribution difference, age-standardized incidence rate, death rate (abbreviated as ASIR and ASDR, respectively), and DALY rate were also used. To track the dynamic changes of disease burden, we used a statistical model termed estimated annual percentage changes (EAPCs), which was calculated based on ASRs following the formula below: *y *=* α *+* βx*. In this formula, x refers to year and y presents log10 (ASRs). Then, EAPC values could be obtained by EAPC = 100*(10^β−1). If the EAPC value and its 95% CI are above zero, the corresponding change trend will be upwards, and vice versa. Besides, by Pearson’s correlation test model, we analyzed the strengths of correlation between SDI values and ASRs.

### Data visualization

Data visualization was performed by R software (version: 3.6.0). The burden of kidney cancer in different countries or territories was presented via world maps. In this study, packages maps, ggplot2, and dplyr were used.

## Results

### Kidney cancer’s incidence, incidence rate, and change trends

**Fig. 1:**
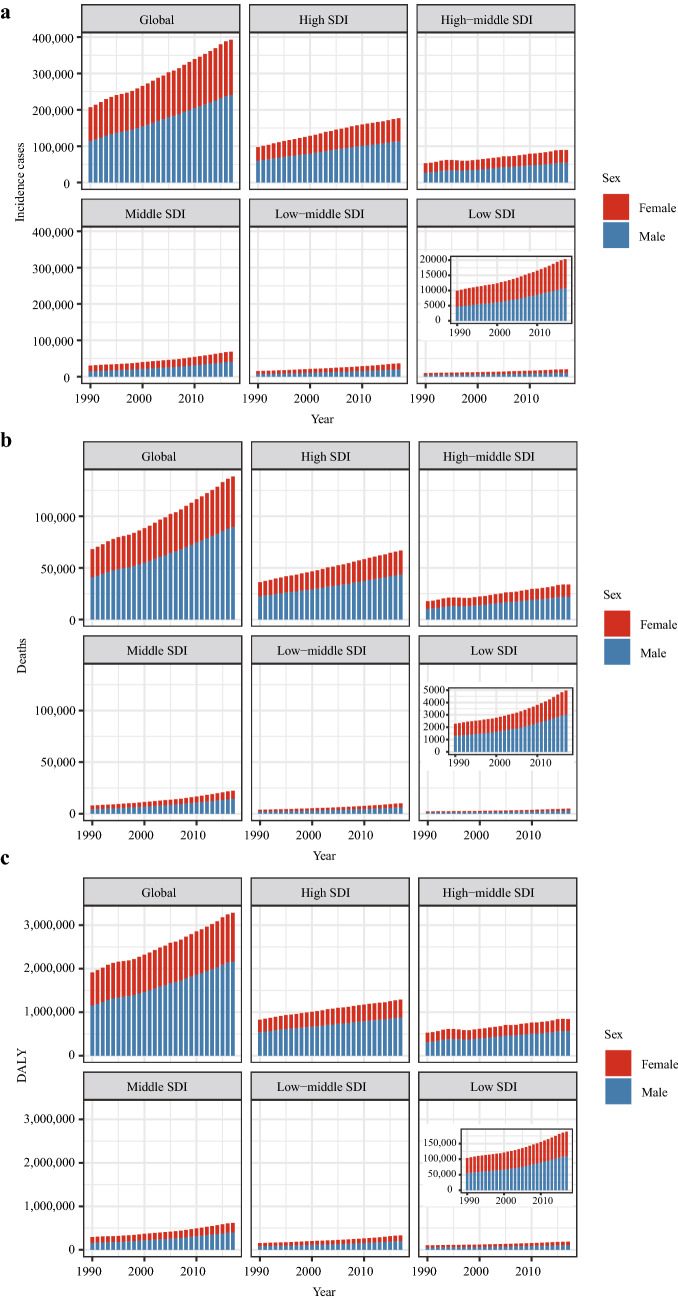
The change trends of kidney cancer’s incidence cases, deaths, and DALYs from 1990 to 2017. **a** The change trends of incidences; **b** the change trends of deaths; **c** the change trends of DALYs. Note: DALY, disability-adjusted life year

**Fig. 2:**
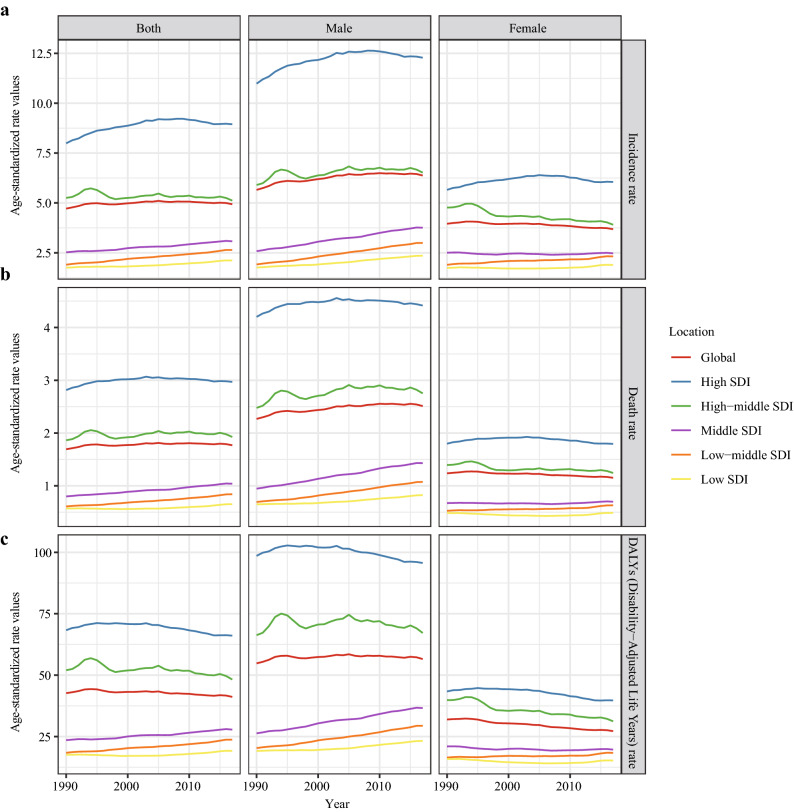
The change trends of kidney cancer’s ASIR, ASDR, and age-standardized DALY rate from 1990 to 2017. **a** The change trends of ASIR; **b** the change trends of ASDR; **c** the change trends of age-standardized DALY rate. Note: ASIR, Age-standardized incidence rate; ASDR, Age-standardized death rate; DALY, disability-adjusted life year

**Fig. 3:**
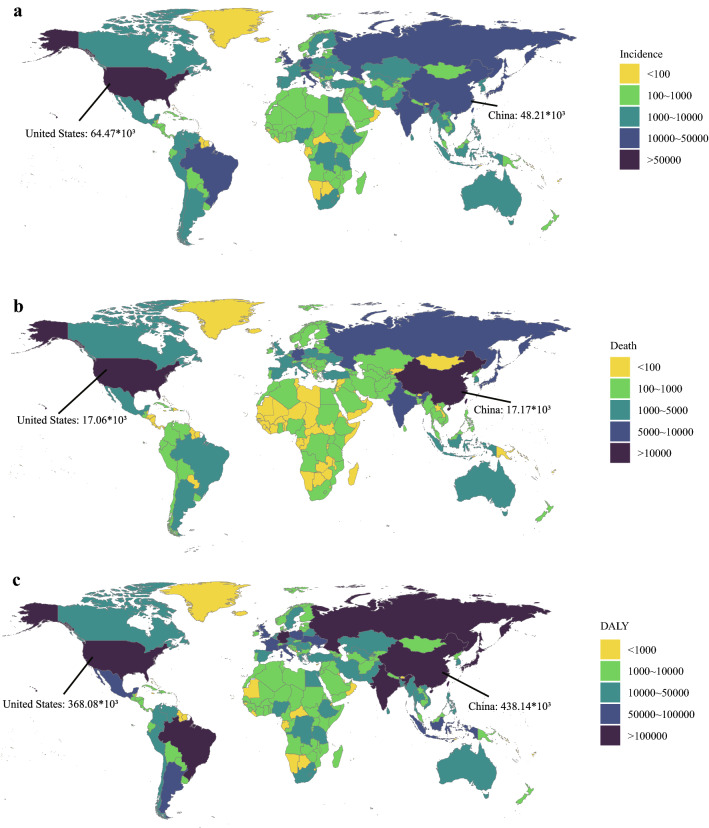
The global disease burden of kidney cancer in 195 countries or territories. **a** The incidence cases of 195 countries in 2017; **b** the deaths of 195 countries in 2017; **c** the DALYs of 195 countries in 2017. Note: DALY, disability-adjusted life year

**Fig. 4:**
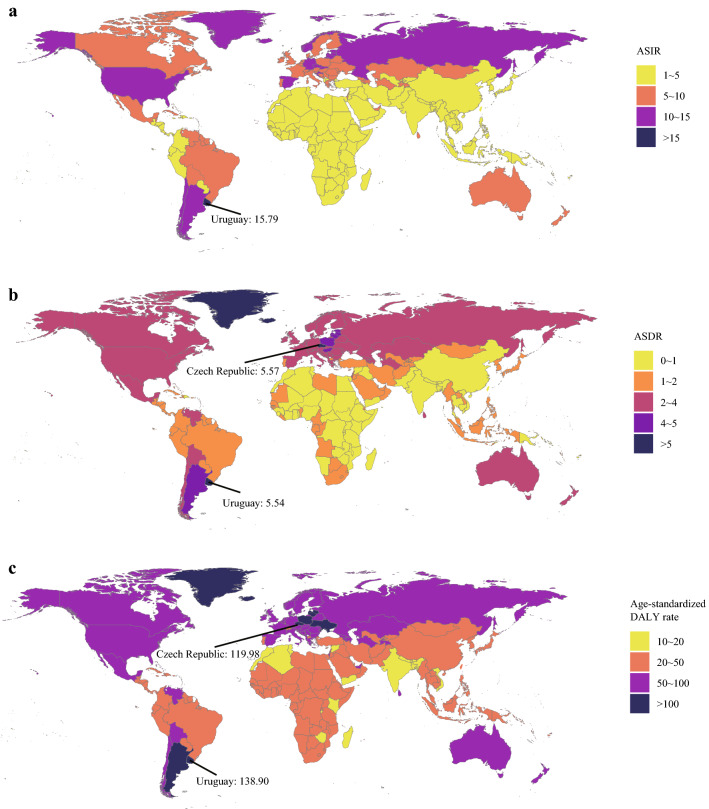
The age-standardized rates of kidney cancer in 195 countries or territories. **a** The ASIR of 195 countries in 2017; **b** the ASDR of 195 countries in 2017; **c** the age-standardized DALY rate of 195 countries in 2017. Note: ASIR, Age-standardized incidence rate; ASDR, Age-standardized death rate; DALY, disability-adjusted life year

From a global perspective, the incidence case of kidney cancer increased sharply from 207.31*10^3^ in 1990 to 393.04*10^3^ in 2017 (Table 1). Kidney cancer mainly occurred in males (the ratio of male patients to female patients: 1.24:1 in 1990 and 1.58:1 in 2017). Offsetting the differences in population size and age distribution between males and females, males indeed had a higher risk of developing kidney cancer than females (the ratio of male’s ASIR to female’s ASIR: 1.43 in 1990 and 1.73 in 2017). At the same time, the growth speed of incidence rate in males was faster than in females (EAPC of male: 0.43, 95% CI 0.34–0.51; EAPC of female: − 0.31, 95% CI − 0.36 to − 0.26). In terms of social development degree, the incidence and ASIR were significantly higher in high SDI countries (Figs. 1a and 2a). Notably, the incidence rate elevated rapidly in low-middle SDI countries (EAPC: 1.21, 95% CI 1.18–1.24). Then, for different geographic areas, Western Europe had the most incidence cases (incidence: 44.01*10^3^ in 1990 and 72.67*10^3^ in 2017), and the high-income North America zone had the highest ASIR (ASIR: 11.74 in 1990 and 12.15 in 2017). South Asia had the fastest increase in ASIR during the past 28 years (EAPC: 1.39, 95% CI 1.32–1.46). As for a specific country, the United States and China had the most incidence cases in 2017 (United States: 64.47*10^3^; China: 48.21*10^3^) (Fig. 3a). Uruguay and Slovakia had the highest ASIR in 2017 (Uruguay: 15.79; Slovakia: 14.14) (Fig. 4a).

**Table 1:** The incidence of kidney cancer in 1990/2017 and temporal trends

	1990	1990	2017	2017	1990–2017
	Incident cases No *10^3^ (95% CI)	ASIR/100,000 No. (95% CI)	Incident cases No *10^3^ (95% CI)	ASIR/100,000 No. (95% CI)	EAPC No. (95% CI)
Overall	207.31 (189.04–219.53)	4.72 (4.29–4.95)	393.04 (371.16–404.59)	4.94 (4.66–5.08)	0.14 (0.07–0.21)
Sex					
Male	114.61 (109.03–120.75)	5.65 (5.40–5.95)	240.77 (225.64–248.69)	6.38 (5.98–6.58)	0.43 (0.34–0.51)
Female	92.7 (75.61–104.74)	3.96 (3.21–4.42)	152.27 (140.67–157.89)	3.68 (3.4–3.82)	− 0.31(− 0.36–− 0.26)
Socio-demographic factor					
High SDI	97.57 (87.7–100.13)	7.99 (7.18–8.20)	176.88 (167.41–182.88)	8.94 (8.55–9.25)	0.37 (0.25–0.50)
High-middle SDI	53.04 (47.18–57.11)	5.25 (4.69–5.65)	89.42 (84.92–93.56)	5.12 (4.87–5.35)	− 0.15 (− 0.26–− 0.04)
Middle SDI	30.81 (28.64–33.34)	2.53 (2.38–2.73)	68.75 (64.39–73.00)	3.08 (2.89–3.27)	0.77 (0.73–0.81)
Low-middle SDI	15.41 (12.63–18.59)	1.90 (1.65–2.21)	36.59 (33.82–39.34)	2.64 (2.44–2.82)	1.21 (1.18–1.24)
Low SDI	9.93 (6.29–14.41)	1.75 (1.31–2.36)	20.35 (17.44–23.30)	2.12 (1.80–2.39)	0.68 (0.60–0.76)
Region					
Andean Latin America	1.37 (1.16–1.55)	4.75 (4.00–5.23)	2.83 (2.46–3.16)	5.05 (4.39–5.64)	0.06 (− 0.15–0.27)
Australasia	1.61 (1.44–1.69)	6.86 (6.17–7.19)	3.87 (3.49–4.32)	8.79 (7.90–9.80)	0.85 (0.59–1.11)
Caribbean	1.87 (1.38–2.31)	6.11 (4.51–7.35)	2.31 (2.05–2.81)	4.67 (4.13–5.67)	− 0.95 (− 1.36–− 0.53)
Central Asia	3.06 (2.56–3.56)	5.59 (4.62–6.60)	5.18 (4.84–5.52)	6.28 (5.87–6.67)	0.22 (0.09–0.35)
Central Europe	10.2 (9.64–10.58)	6.84 (6.47–7.10)	17.17 (14.77–18.05)	8.65 (7.46–9.07)	1.12 (0.92–1.32)
Central Latin America	5.03 (4.47–5.25)	4.42 (3.99–4.58)	13.76 (13.07–14.58)	5.68 (5.40–6.02)	1.01 (0.94–1.08)
Central Sub-Saharan Africa	1.04 (0.61–1.65)	2.36 (1.85–3.08)	2.19 (1.76–2.73)	2.66 (2.19–3.27)	0.36 (0.27–0.45)
East Asia	25.17 (22.57–30.08)	2.30 (2.06–2.77)	52.29 (46.83–56.23)	2.77 (2.49–2.98)	1.11 (0.91–1.31)
Eastern Europe	23.77 (20.36–26.69)	8.55 (7.36–9.57)	32.27 (30.31–33.74)	10.02 (9.49–10.47)	0.30 (0.07–0.54)
Eastern Sub-Saharan Africa	3.76 (2.21–5.98)	2.29 (1.64–3.34)	6.91 (5.66–8.39)	2.47 (2.11–2.87)	0.13 (0.05–0.22)
High-income Asia Pacific	6.61 (6.26–7.11)	3.27 (3.10–3.53)	16.89 (14.91–18.25)	4.42 (3.94–4.83)	1.14 (0.85–1.43)
High-income North America	39.47 (35.50–40.63)	11.74 (10.54–12.11)	68.84 (65.66–74.2)	12.15 (11.55–13.19)	− 0.05 (− 0.17–0.06)
North Africa and Middle East	6.21 (4.73–7.98)	2.45 (1.97–2.98)	15.08 (12.98–16.23)	3.09 (2.68–3.31)	1.04 (0.94–1.13)
Oceania	0.11 (0.09–0.15)	2.66 (2.06–3.42)	0.27 (0.20–0.35)	2.91 (2.27–3.75)	0.38 (0.34–0.42)
South Asia	10.18 (7.98–13.05)	1.26 (1.04–1.55)	27.98 (25.44–29.68)	1.87 (1.70–1.97)	1.39 (1.32–1.46)
Southeast Asia	9.13 (7.52–10.77)	2.70 (2.34–3.09)	20.83 (17.71–22.72)	3.32 (2.84–3.61)	0.56 (0.35–0.77)
Southern Latin America	5.82 (4.14–6.35)	12.13 (8.62–13.24)	9.10 (8.14–10.17)	11.61 (10.39–13.02)	− 0.11 (− 0.39–0.18)
Southern Sub-Saharan Africa	0.99 (0.88–1.1)	2.62 (2.31–2.94)	2.06 (1.830–2.29)	3.28 (2.93–3.63)	0.81 (0.3–1.32)
Tropical Latin America	4.45 (4.03–4.7)	3.70 (3.38–3.87)	11.70 (11.10–12.22)	5.04 (4.79–5.26)	1.16 (1.11–1.22)
Western Europe	44.01 (38.63–45.65)	8.13 (7.16–8.44)	72.67 (65.48–76.76)	9.16 (8.27–9.69)	0.43 (0.33–0.53)
Western Sub-Saharan Africa	3.44 (2.57–4.52)	2.19 (1.77–2.72)	8.84 (7.40–10.33)	2.92 (2.52–3.41)	0.91 (0.8–1.02)

### Kidney cancer’s death, death rate, and change trends

In the globe, the deaths of kidney cancer increased from 68.14*10^3^ in 1990 to 138.53*10^3^ in 2017 (Table 2) (Fig. 1b). Subgroup analysis by sex showed that males contributed more to the mushrooming kidney cancer-related deaths than females (total death number of males in 2017: 89.62*10^3^; total death number of females in 2017: 48.91*10^3^; EAPC of males: 0.37, 95% CI 0.31–0.44; EAPC of females: − 0.32, 95% CI − 0.37 to − 0.28). Subgroup analysis by SDI values showed that kidney cancer-related death mainly occurred in high SDI countries (death cases: 36.13*10^3^ in 1990 and 66.86*10^3^ in 2017; ASDR: 2.81 in 1990 and 2.97 in 2017). At the same time, the ASDR was rapidly elevated in low-middle SDI countries (ASDR: 0.61 in 1990 and 0.84 in 2017; EAPC: 1.20, 95% CI 1.16–1.23) (Fig. 2b). Subgroup analysis by geographic area demonstrated that Western Europe had the most kidney cancer-related deaths (18.58*10^3^ in 1990 and 30.33*10^3^ in 2017), and Southern Latin America had the highest ASDR (4.61 in 1990 and 4.28 in 2017). Besides, the growth speed of ASDR in East Asia was fastest (EAPC: 2.01, 95% CI 1.78–2.23). In the level of country or territory, China and the United States had the most kidney cancer-related deaths in 2017 (17.17*10^3^ and 17.06*10^3^, respectively) (Fig. 3b). Besides, the Czech Republic and Uruguay had the highest ASDR in 2017 (Czech Republic: 5.57; Uruguay: 5.54) (Fig. 4b).

**Table 2:** The death of kidney cancer in 1990/2017 and temporal trends

	1990	1990	2017	2017	1990–2017
	Death cases No *10^3^ (95% CI)	ASDR/100,000 No. (95% CI)	Death cases No *10^3^ (95% CI)	ASDR/100,000 No. (95% CI)	EAPC No. (95% CI)
Overall	68.14 (62.73–70.8)	1.69 (1.56–1.75)	138.53 (128.66–142.52)	1.77 (1.64–1.82)	0.13(0.07–0.19)
Sex					
Male	40.97 (39.1–42.75)	2.27 (2.17–2.36)	89.62 (84.64–92.52)	2.51 (2.38–2.59)	0.37 (0.31–0.44)
Female	27.17 (21.98–29.44)	1.24 (1–1.33)	48.91 (42.92–50.65)	1.15 (1.01–1.19)	− 0.32 (− 0.37–− 0.28)
Socio-demographic factor					
High SDI	36.13 (32.66–36.86)	2.81 (2.54–2.87)	66.86 (60.67–69.15)	2.97 (2.73–3.07)	0.13 (0.04–0.21)
High-middle SDI	17.74 (16.05–18.97)	1.86 (1.7–1.99)	33.90 (31.85–35.32)	1.93 (1.81–2.01)	0.11 (− 0.01–0.22)
Middle SDI	8.00 (7.53–8.66)	0.80 (0.75–0.87)	22.32 (20.93–23.78)	1.04 (0.98–1.11)	0.99 (0.96–1.02)
Low-middle SDI	3.84 (3.36–4.46)	0.61 (0.53–0.69)	10.08 (9.36–10.77)	0.84 (0.78–0.90)	1.20 (1.16–1.23)
Low SDI	2.29 (1.7–3.15)	0.57 (0.43–0.73)	4.97 (4.21–5.55)	0.65 (0.55–0.73)	0.47 (0.34–0.60)
Region					
Andean Latin America	0.37 (0.33–0.40)	1.6 (1.39–1.72)	0.98 (0.84–1.09)	1.82 (1.56–2.03)	0.36 (0.17–0.54)
Australasia	0.79 (0.69–0.82)	3.27 (2.86–3.4)	1.59 (1.44–1.74)	3.27 (2.97–3.59)	− 0.19 (− 0.25–− 0.13)
Caribbean	0.54 (0.41–0.61)	1.94 (1.48–2.16)	0.76 (0.70–0.90)	1.51 (1.39–1.77)	− 0.88 (− 1.29–− 0.48)
Central Asia	0.91 (0.75–1.08)	1.79 (1.46–2.14)	1.70 (1.61–1.79)	2.24 (2.13–2.36)	0.66 (0.52–0.79)
Central Europe	4.20 (4.02–4.31)	2.78 (2.66–2.86)	8.10 (7.04–8.5)	3.81 (3.32–3.99)	1.37 (1.14–1.6)
Central Latin America	1.50 (1.36–1.54)	1.59 (1.46–1.64)	4.54 (4.31–4.80)	1.95 (1.85–2.06)	0.78 (0.71–0.85)
Central Sub-Saharan Africa	0.23 (0.18–0.31)	0.79 (0.61–0.97)	0.49 (0.41–0.60)	0.85 (0.68–1.11)	0.15 (− 0.04–0.33)
East Asia	6.05 (5.43–7.66)	0.65 (0.58–0.84)	18.63 (16.49–19.99)	0.97 (0.86–1.04)	2.01 (1.78–2.23)
Eastern Europe	8.81 (7.75–9.79)	3.07 (2.71–3.4)	12.95 (12.18–13.37)	3.78 (3.58–3.9)	0.56 (0.3–0.82)
Eastern Sub-Saharan Africa	0.77 (0.54–1.15)	0.73 (0.57–0.98)	1.47 (1.26–1.70)	0.77 (0.67–0.88)	0.04 (− 0.06–0.14)
High-income Asia Pacific	3.08 (3.00–3.29)	1.53 (1.49–1.64)	8.97 (7.76–9.52)	1.91 (1.69–2.03)	0.78 (0.57–0.99)
High-income North America	11.12 (10.08–11.36)	3.14 (2.85–3.21)	19.05 (18.30–20.09)	3.11 (2.98–3.29)	− 0.26 (− 0.36–− 0.16)
North Africa and Middle East	1.67 (1.37–2.03)	0.85 (0.69–0.99)	4.50 (3.91–4.82)	1.07 (0.93–1.15)	1.06 (0.92–1.2)
Oceania	0.02 (0.02–0.03)	0.75 (0.58–0.97)	0.06 (0.05–0.07)	0.85 (0.68–1.08)	0.5 (0.45–0.54)
South Asia	2.82 (2.33–3.46)	0.45 (0.36–0.54)	8.32 (7.51–8.8)	0.62 (0.56–0.66)	1.16 (1.03–1.28)
Southeast Asia	2.10 (1.86–2.36)	0.77 (0.69–0.86)	5.97 (5.10–6.47)	1.04 (0.89–1.12)	0.79 (0.53–1.05)
Southern Latin America	2.19 (1.64–2.31)	4.61 (3.46–4.85)	3.49 (3.19–3.86)	4.28 (3.9–4.72)	− 0.28 (− 0.52–− 0.05)
Southern Sub-Saharan Africa	0.28 (0.24–0.31)	0.91 (0.78–1.04)	0.63 (0.56–0.69)	1.13 (1.01–1.24)	0.83 (0.48–1.18)
Tropical Latin America	1.29 (1.2–1.34)	1.29 (1.21–1.34)	4.03 (3.81–4.21)	1.76 (1.67–1.84)	1.17 (1.13–1.21)
Western Europe	18.58 (16.37–19.09)	3.16 (2.78–3.24)	30.33 (27.10–31.84)	3.26 (2.95–3.43)	0.06 (0.02–0.1)
Western Sub-Saharan Africa	0.81 (0.65–1.00)	0.73 (0.61–0.88)	1.96 (1.70–2.27)	0.91 (0.79–1.06)	0.72 (0.63–0.81)

### Kidney cancer’s DALY, DALY rate, and change trends

In the global level, the kidney cancer-related DALYs were increased from 1915.49*10^3^ in 1990 to 3284.32*10^3^ in 2017 (Table 3) (Fig. 1c). Most DALYs were contributed by males (DALYs of males in 1990: 1157.59*10^3^; DALY in 2017: 2166.29*10^3^). Subgroup analysis by SDI values showed that high SDI countries had the most DALYs (828.98*10^3^ in 1990 and 1289.66*10^3^ in 2017). Low-middle SDI countries had the fastest rise in age-standardized DALY rate at the same time (EAPC = 0.93, 95% CI 0.89–0.96) (Fig. 2c). For different geographic areas, Western Europe had the highest DALY burden (405.97*10^3^ in 1990 and 553.19*10^3^ in 2017). At the same time, East Asia had the most rapid increase in age-standardized DALY rate (age-standardized DALY rate: 20.84 in 1990 and 25.15 in 2017, EAPC = 1.13, 95% CI 0.92–1.33). In the level of country or territory, China and the United States had the highest DALY burden in 2017 (438.14*10^3^ and 368.08*10^3^, respectively) (Fig. 3c). Besides, Uruguay and the Czech Republic had the highest age-standardized DALY rate in 2017 (138.90 and 119.98, respectively) (Fig. 4c).

**Table 3:** The DALY of kidney cancer in 1990/2017 and temporal trends

	1990	1990	2017	2017	1990–2017
	DALY No *10^3^ (95% CI)	Average standardized DALY rate/100,000 No. (95% CI)	DALY No *10^3^ (95% CI)	Average standardized DALY rate/100,000 No. (95% CI)	EAPC No. (95% CI)
Overall	1915.49 (1735.86–2037.43)	42.66 (38.85–44.93)	3284.32 (3085.56–3393.16)	41.14 (38.66–42.49)	− 0.20 (− 0.25–− 0.14)
Sex					
Male	1157.59 (1093.79–1226.54)	54.80 (52.05–57.55)	2166.29 (2026.86–2241.74)	56.55 (52.96–58.47)	0.07 (0.003–0.13)
Female	757.89 (611.81–865.34)	31.94 (25.65–35.98)	1118.03 (1030.18–1163.02)	27.21 (25.03–28.29)	− 0.67 (− 0.71–− 0.63)
Socio-demographic factor					
High SDI	828.98 (750.18–851.84)	68.29 (61.81–70.16)	1289.66 (1214.29–1335.62)	66.08 (62.70–68.45)	− 0.23 (− 0.32–− 0.15)
High-middle SDI	529 (469.86–569.13)	52.00 (46.34–55.91)	843.42 (798.96–879.28)	48.22 (45.83–50.23)	− 0.34 (− 0.47–− 0.22)
Middle SDI	293.74 (274.47–319.99)	23.55 (22.19–25.50)	620.20 (575.96–660.73)	27.83 (26.06–29.59)	0.66 (0.62–0.71)
Low-middle SDI	154.97 (127.78–187.76)	18.40 (15.93–21.49)	332.52 (305.98–357.67)	23.73 (21.89–25.43)	0.93 (0.89–0.96)
Low SDI	103.7 (66.24–154.98)	17.59 (13.07–24.26)	189.05 (160.88–214.37)	19.20 (16.35–21.54)	0.30 (0.18–0.43)
Region					
Andean Latin America	14.16 (12.44–15.65)	47.50 (41.17–51.57)	25.82 (22.59–28.92)	46.02 (40.19–51.29)	− 0.27 (− 0.47–− 0.06)
Australasia	17.7 (15.63–18.46)	75.79 (67.21–79.05)	31.61 (28.62–34.96)	73.12 (66.28–80.9)	− 0.29 (− 0.34–− 0.24)
Caribbean	18.48 (13.95–22.23)	59.99 (45.5–70.56)	20.95 (18.80–25.11)	42.6 (38.09–51.12)	− 1.22 (− 1.64–− 0.79)
Central Asia	30.12 (25.58–34.93)	53.43 (44.63–63.17)	52.34 (49.29–55.11)	62.42 (58.91–65.65)	0.38 (0.24–0.51)
Central Europe	110.83 (103.52–114.07)	74.80 (69.81–76.99)	175.47 (155.5–184.35)	90.34 (80.08–94.73)	0.88 (0.68–1.09)
Central Latin America	53.16 (47.16–54.97)	45.23 (40.77–46.72)	126.95 (120.72–133.94)	52.44 (49.88–55.37)	0.6 (0.52–0.69)
Central Sub-Saharan Africa	11.10 (6.42–17.06)	24.08 (18.64–31.37)	20.76 (16.72–25.24)	24.47 (20.51–29.51)	− 0.05 (− 0.18–0.09)
East Asia	229.95 (207.43–275.71)	20.84 (18.82–25.49)	472.46 (417.97–507.41)	25.15 (22.46–26.95)	1.13 (0.92–1.33)
Eastern Europe	244.68 (212.02–274.15)	87.5 (75.87–97.79)	318.58 (304.82–330.17)	98.87 (94.69–102.7)	0.18 (− 0.12–0.47)
Eastern Sub-Saharan Africa	40.17 (23.58–65.07)	23.34 (16.67–34.25)	66.59 (54.91–79.91)	22.79 (19.51–26.36)	− 0.23 (− 0.34–− 0.12)
High-income Asia Pacific	72.28 (70.38–76.52)	35.45 (34.47–37.66)	149.03 (134.76–159.4)	39.63 (35.93–42.70)	0.38 (0.18–0.59)
High-income North America	263.79 (238.77–272.98)	79.85 (72.31–82.71)	407.78 (387.52–435.78)	72.6 (68.88–77.99)	− 0.62 (− 0.72–-0.52)
North Africa and Middle East	67.12 (50.45–86.17)	25.30 (20.27–31.00)	140.56 (120.36–150.18)	28.67 (24.82–30.66)	0.65 (0.53–0.77)
Oceania	0.96 (0.74–1.22)	21.72 (17.04–27.9)	2.28 (1.78–2.92)	24.44 (19.16–30.90)	0.50 (0.44–0.56)
South Asia	104.55 (82.2–134.68)	12.59 (10.39–15.56)	251.84 (225.69–265.85)	16.76 (15.01–17.69)	0.98 (0.88–1.09)
Southeast Asia	76.04 (62.63–88.55)	21.92 (19.02–24.93)	176.89 (149.46–192.46)	28.11 (23.79–30.55)	0.72 (0.49–0.95)
Southern Latin America	60.96 (44.73–64.64)	126.5 (92.75–134.2)	80.86 (73.15–90.63)	103.09 (93.24–115.75)	− 0.76 (− 1.07–− 0.45)
Southern Sub-Saharan Africa	10.16 (9.12–11.31)	26.39 (23.39–29.43)	19.10 (16.97–21.29)	30.27 (26.95–33.54)	0.47 (0.02–0.91)
Tropical Latin America	47.28 (43.26–49.74)	38.56 (35.48–40.15)	107.71 (102.44–112.13)	46.71 (44.32–48.56)	0.73 (0.69–0.78)
Western Europe	405.97 (359.81–418.97)	75.35 (67.02–77.76)	553.19 (510.18–582.26)	70.68 (65.64–74.49)	− 0.34 (− 0.39–− 0.29)
Western Sub-Saharan Africa	36.02 (27.06–47.51)	22.05 (17.85–27.27)	83.55 (70.63–98.56)	26.42 (22.8–30.72)	0.50 (0.37–0.63)

### The correlation analysis between SDI value and kidney cancer’s burden

**Fig. 5:**
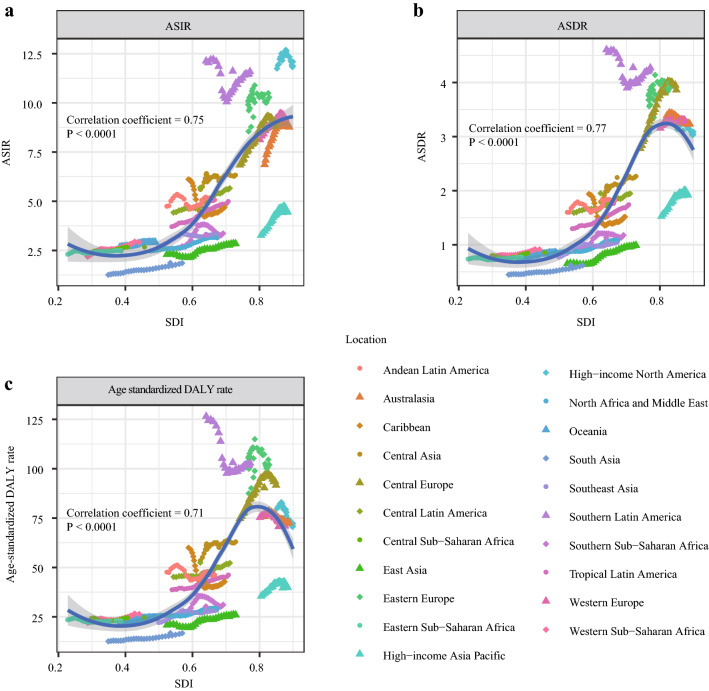
The correlation analyses of ASRs and SDI values from 1990 to 2017. **a** The correlation of ASIR and SDI from 1990 to 2017. **b** The correlation of ASDR and SDI from 1990 to 2017. **c** The correlation of age-standardized DALY rate and SDI from 1990 to 2017. Note: ASIR, Age-standardized incidence rate; ASDR, Age-standardized death rate; DALY, disability-adjusted life year; SDI, Socio-demographic Index

To explore the potential correlation between social development degree and kidney cancer’s burden, we conducted a correlation analysis between SDI values and ASRs in 21 different geographic areas from 1990 to 2017. The results indicated that ASRs were significantly positively correlated to SDI values (coefficient of ASIR-SDI: 0.75, of ASDR-SDI: 0.77, of age-standardized DALY rate-SDI: 0.71; all P values < 0.0001) (Fig. 5a–c).

### Age distribution

**Fig. 6:**
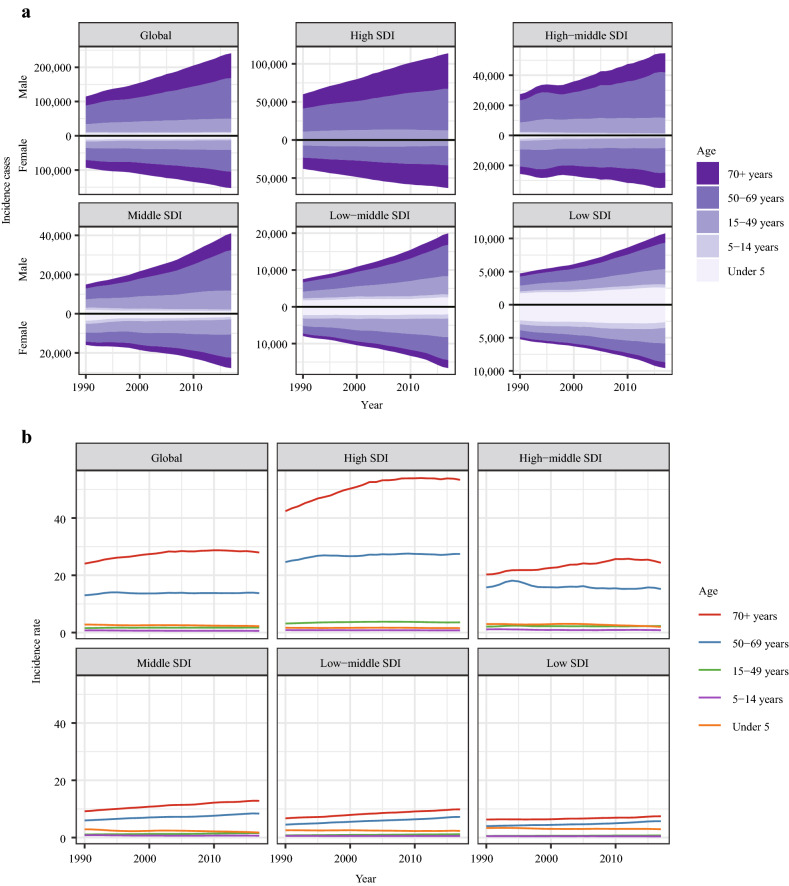
The change trends of incidence and ASIR of kidney cancer in different age groups. **a** The incidence cases of kidney cancer in five different age groups in the globe and in various regions. The ASIR of kidney cancer in five different age groups in the world and in various regions. Note: ASIR, Age-standardized incidence rate; SDI, Socio-demographic Index

Age distribution is a vital parameter of cancer epidemiology. In the globe, nearly 77% of kidney cancer patients aged 50 years or older in 2017 (Fig. 6a). This ratio reached 88% in the high SDI counties. Relatively, the ratio of older patients was lower in low SDI countries (just 45% of patients aged 50 years or older). Notably, for the age group under 5 years, kidney cancer’s incidence in males and females was roughly similar. However, for the age group above 70 years or older, male patients far outnumbered female patients in 2017 (male patients: 72.61*10^3^ and female patients: 48.43*10^3^). Generally, in countries with different SDI values, the ASIR was highest in the patients aged 70 years or older (Fig. 6b).

### Attributable risk factors

**Fig. 7:**
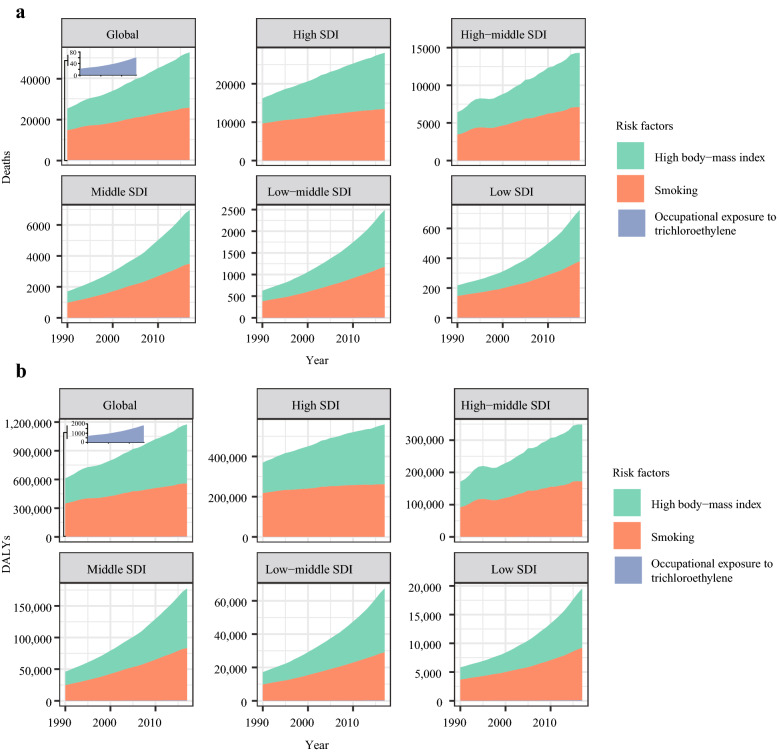
Risk factors contributing to kidney cancer-related death and DALY. **a** Three risk factors contributing to kidney cancer-related death from 1990 to 2017 in the globe and different regions; **b** three risk factors contributing to AML-related DALY from 1990 to 2017 in the world and different regions; Note: DALY, disability-adjusted life year; SDI, Socio-demographic Index

We searched the GBD database for risk factors contributing to kidney cancer-related mortality. Eventually, we found that high body-mass index, smoking, occupational exposure to trichloroethylene were mainly attributable risk factors of kidney cancer-related mortality. Among them, high body-mass index and smoking were leading risk factors of kidney cancer-related death and DALY (Fig. 7a, b). The contribution ratio of occupational exposure to trichloroethylene was relatively weak.

## Discussion

In this study, we comprehensively analyzed the burden of kidney cancer and attributable risk factors. A previous GLOBOCAN-based cancer epidemiologic study showed that kidney cancer’s incidence rate kept increasing in most countries [33]. In some high-income developed areas such as the United States, Western and Northern Europe, and Australia, the kidney cancer-related mortality rate kept a high level in the globe but began to decline from its peak value recently [33]. Contrarily, in Africa and most Asian countries, the incidence rate of kidney cancer was relatively low but increased rapidly in the past decades [33]. Similarly to the results of a GLOBOCAN database-based study, our study showed SDI value was significantly positively correlated to ASIR, ASDR, and age-standardized DALY rate. This increased ASIR in high SDI countries could be attributed to multiple factors, which included but were not limited to cancer registry system, aging, smoking, obesity, and hypertension [34]. Besides, more widely used abdominal cross-sectional imaging technology also contributes to a larger number of incidental diagnosis of kidney cancer in developed countries [35]. Notably, although both incidence rate and mortality rate were higher in developed countries, the mortality-to-incidence ratio was lower in these countries simultaneously [35]. This disparity might closely associate with the quality and availability of health care [35].

In the context of the aging in the globe, the increasing trend of kidney cancer burden is likely to continue in the future. Therefore, policy-makers need to take action to relieve this trend. Decreasing the influence of some attributable risk factors such as high body-mass index and smoking is feasible to alleviate the growing burden of kidney cancer. The correlation between obesity and kidney cancer has been a concern for a long time [36]. A previously retrospective study showed that each additional body-mass index value increased the relative risk ratio of kidney cancer by 7% [37]. Later, another perspective study proved that a high body-mass index was an independent prognostic factor for kidney cancer (Hazard Ratio = 1.71) [38]. The mechanisms by which obesity affects the incidence and survival of kidney cancer might include insulin/insulin-like growth factor signals, chronic inflammation, sex steroids, as well as the treatment disparities between obese patients and normal-weight patients [39].

Tobacco smoking is another vital risk factor contributing to kidney cancer-related mortality. A previous meta-analysis showed that smoking markedly increased the risk of kidney cancer’s incidence and disease-specific mortality [40]. The biologic mechanisms by which smoking induces and promotes kidney cancer are still unclear [41]. However, smoking could certainly cause renal damage by multiple manners, including oxidative stress, tubulotoxic effect, endothelial cell dysfunction, and hemodynamic change [42]. These cytotoxic activities increase cell turnover and lead to DNA damage, which might involve in cancer initiation and progression [42]. Additionally, smoking is closely related to some genetic or epigenetic alterations such as gene mutations and methylations [42]. Therefore, it is essential to emphasize the necessity of smoking cessation to the public to minimize kidney cancer’s burden.

In the GBD database, we observed that occupational exposure to trichloroethylene was also a contributor to kidney cancer-related mortality. It has been reported that the relationship between trichloroethylene and kidney cancer risk is dose–response with trichloroethylene exposure level [4]. Kidney is the main target of trichloroethylene [43]. The nephrotoxic and potential nephrocarcinogenic effects of trichloroethylene occur predominantly by GST conjugation and bio-activation by renal CCLB1 [44]. Protection against occupational exposure is necessary, especially for individuals in the organic/chlorinated solvent industry.

Generally, developed countries with high SDI had the highest kidney cancer’s burden but had a slight decrease in incidence rate. On the contrary, the incidence rate was rapidly increased in developing countries with low-middle SDI countries, although the burden of kidney cancer kept relatively low until 2017. The elderly population was the group with the highest risk of developing kidney cancer. With more countries facing an aging population, it is important to be aware of the potentially increased burden of kidney cancer. Lastly, attributable risk factor analysis showed that the high body-mass index and smoking were the main factors contributing to kidney cancer-related mortality. Reinforcing a healthy lifestyle to the public would be helpful to minimize kidney cancer’s burden in the future.

## Conclusion

In the present study, we performed a comprehensive analysis to assess the burden of kidney cancer in the globe, different areas, and 195 countries from 1990 to 2017. The incidence of kidney cancer kept growing in the past 28 years. Generally, developed countries with high SDI had the highest kidney cancer’s burden but had a slight decrease in incidence rate. On the contrary, the incidence rate was rapidly increased in developing countries with low-middle SDI countries, although the burden of kidney cancer kept relatively low until 2017. The elderly population was the group with the highest risk of developing kidney cancer. With more countries facing an aging population, it is important to be aware of the potentially increased burden of kidney cancer. Lastly, attributable risk factor analysis showed that the high body-mass index and smoking were the main factors contributing to kidney cancer-related mortality. Reinforcing a healthy lifestyle to the public would be helpful to minimize kidney cancer’s burden in the future.

## Data Availability

The datasets generated during and/or analyzed during the current study are available from the Global Health Data Exchange query tool (http://ghdx.healthdata.org/gbd-results-tool).

## Abbreviations

GBD: Global burden disease
DALY: Disability-adjusted life year
SDI: Socio-demographic Index
ASIR: Age-standardized incidence rate
ASDR: Age-standardized death rate
EAPC: Estimated annual percentage change
ASR: Age-standardized rate
